# Cutaneous Reaction as a Test of Pregnacy

**Published:** 1914-08

**Authors:** 


**Cutaneous Reaction as a Test of Pregnacy.** Ernest Engelhorn and Hermann Wintz of Erlangen, Münch. Med. Woch., Mch. 31, 1914, employ a placental extract (method not stated except that it is complicated) in the same way as the von Pirquet tuberculin reaction. The reaction is practically the same. It proved positive in all of 70 pregnant women, from the second month to term. It was negative in 13 men. It was positive in a child of 6 with cystitis and faintly positive in three women just before the menses occurred, otherwise negative in 41 females and children. The test becomes negative shortly after parturition.

